# Unveiling the Extraordinary: A Rare Type IIIA Infra-Popliteal Arterial Variation in a Free Fibula Osteocutaneous Flap for Mandibular Reconstruction

**DOI:** 10.7759/cureus.59984

**Published:** 2024-05-09

**Authors:** Akash Doshi, Firoz Borle, Nitin Bhola

**Affiliations:** 1 Oral and Maxillofacial Surgery, Sharad Pawar Dental College, Datta Meghe Institute of Higher Education and Research, Wardha, IND; 2 Plastic and Reconstructive Surgery, Jawaharlal Nehru Medical College, Datta Meghe Institute of Higher Education and Research, Wardha, IND

**Keywords:** kim-lippert’s classification, infra-popliteal arterial variation, microvascular surgery, head and neck reconstruction, free fibula flap

## Abstract

The free fibula flap (FFF), based on the peroneal artery (PA) system, is the gold standard for mandibular reconstruction. Various anatomical variations in the infra-popliteal lower limb vascular system exist. These variations present as an intraoperative surprise to surgeons even after an unremarkable clinical vascular examination of the leg. Here, we report one such case, where we performed successful mandibular reconstruction after encountering a Type IIIA variation of infra-popliteal arterial vasculature.

## Introduction

The free fibula flap (FFF) is the flap of choice for mandibular reconstruction following segmental resection for oral malignancy [1]. The advantages of a fibula osteocutaneous flap are as follows: a) The fibula is a non-weight-bearing dispensable bone; b) it has a segmental blood supply, allowing for multiple osteotomies; c) it has good bone stock for future dental rehabilitation; d) it can provide a skin paddle for lining; e) it can be harvested simultaneously along with resection of the tumor; and f) it has reliable vascular anatomy [2]. In resource-constrained settings, clinical examination of the peripheral pulses suffices, and imaging for the vascular anatomy is considered optional [3]. However, there are situations where the surgeon can be surprised by the variation in the infra-popliteal vasculature despite a normal clinical examination preoperatively. Knowing these variations is essential to mitigating such situations without compromising the reconstruction and the vascularity of the lower limb. Here, we report one such case where we encountered a Kim-Lippert’s Type IIIA vascular variation of the infra-popliteal arterial branching and performed a successful reconstruction of the mandibular defect.

## Case presentation

**Figure 1:**
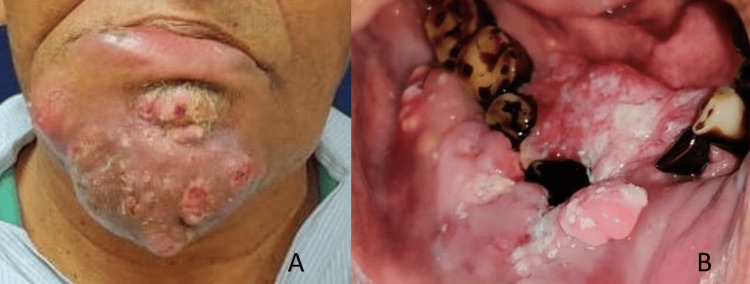
Clinical images of the patient A: Clinical photo showing extraoral fungation. B: Intraoral lesion

**Figure 2:**
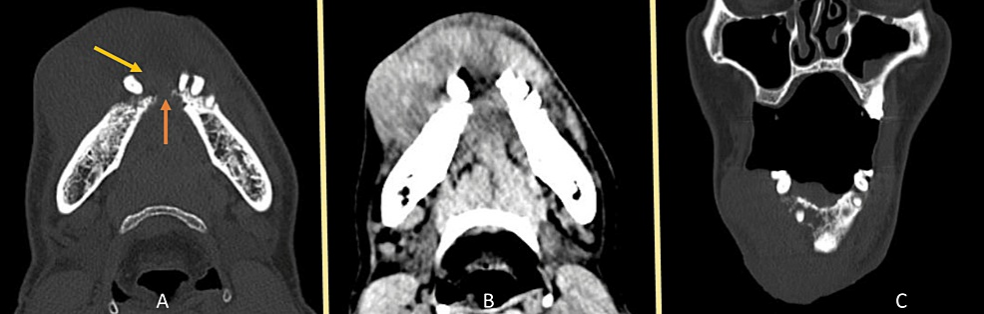
Contrast-enhanced CT scan of the patient's oral lesion A: Hard tissue axial CT scan section of the patient showing erosion of the buccal cortical plate (yellow arrow) and lingual cortical plate (orange arrow). B: Soft tissue axial CT scan section of the patient demonstrating the tumor's spread from the lower right anterior gingivobuccal sulcus to the left gingivobuccal sulcus, with involvement of the skin. C: Coronal CT section revealing bony erosion and the extent of tumor spread

A 55-year-old male presented with ulcero-proliferative growth in the lower gingivobuccal sulcus with an extraoral function of 3.3 x 5.8 x 1.2 cm over the chin region (Figure 1A, 1B). Clinically, there was enlargement of bilateral level 1a and 1b neck nodes. The CT scan confirmed the findings of a lesion extending from the right anterior gingivobuccal sulcus, crossing the midline, and reaching the left gingivobuccal sulcus (Figure 2A-C). The lesion showed erosion of the outer and inner cortex on the right body of the mandible (Figure 2A-C). An incisional biopsy revealed well-differentiated squamous cell carcinoma upon histopathological examination. A high-resolution CT (HRCT) thorax was conducted to rule out lung metastasis. The lesion was classified as TNM stage T4aN2cMx.

A mid-segmental mandibulectomy and an excision of the involved mucosa and extraoral lesion were planned. Bilateral modified radical neck dissection (MRND) for regional control was also planned. The left FFF and free antero-lateral thigh (ALT) flap were planned for mandibular and extraoral reconstruction, respectively. The clinical examination of the left lower limb was unremarkable. The left dorsalis pedis and posterior tibial pulses were palpable and normovolemic. Routinely, we do not perform any imaging if the clinical examination is unremarkable due to financial constraints. Hence, no imaging of the left lower limb vasculature was done.

**Figure 3:**
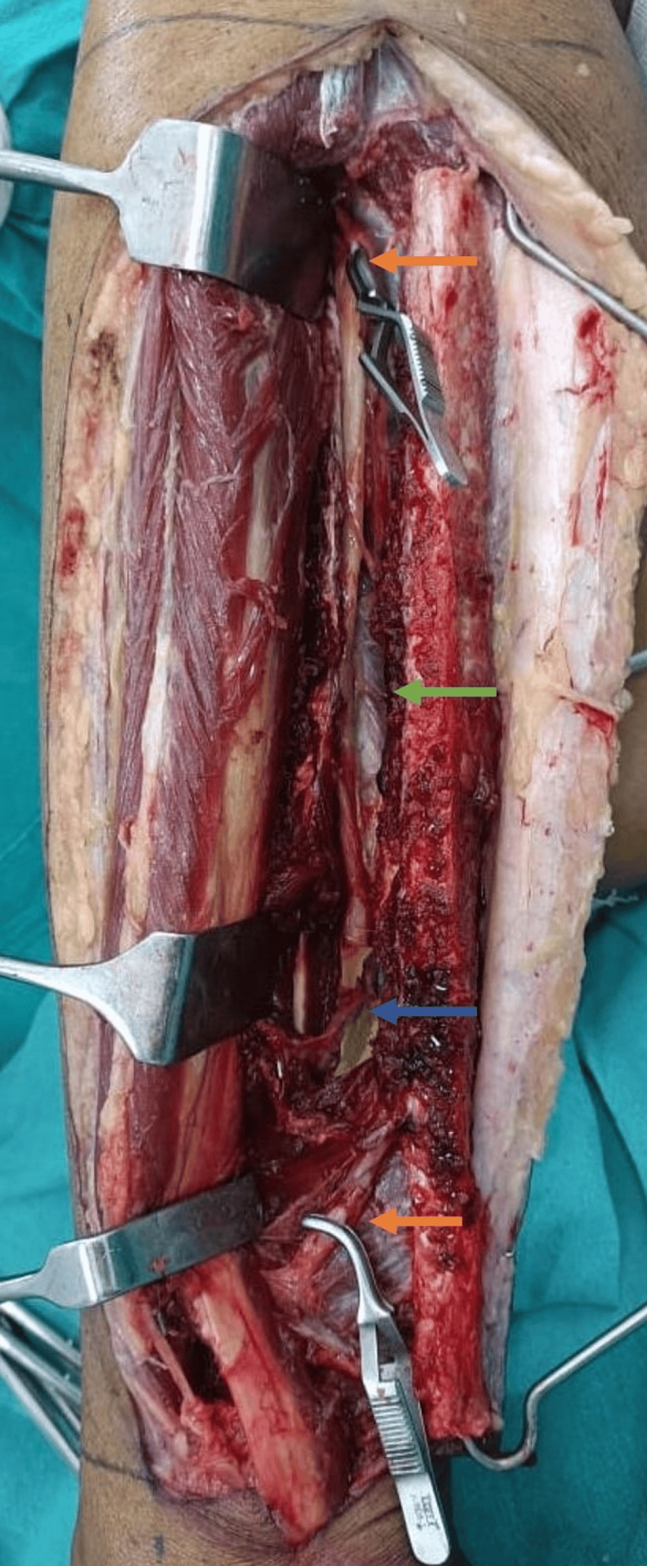
Intraoperative image while harvesting the free fibula flap Clamped peroneal artery both proximally and distally (orange arrow), peroneal vessel (green arrow), and showing distal branching from the peroneal artery (blue arrow)

The extraction of the fibula osteocutaneous flap was started simultaneously with the anterior approach. The peroneal muscles and the extensor muscles were separated from the fibula. The anterior tibial vessels of normal caliber were present. The interosseous membrane and the tibialis posterior muscle were incised to expose the peroneal vessels. Before dividing the distal end of the peroneal vessels, we tried to confirm the presence of the posterior tibial artery (PTA). We found that the PTA was missing proximally. There was a low bifurcation arising from peroneal vessels about 10 cm proximal to the lateral malleolus. Continuing the harvest could have resulted in a vascular compromise of the left lower limb. Hence, bulldog atraumatic vascular clamps were placed proximally and over the distal runoff of the peroneal artery (PA) (Figure 3).

The tourniquet was deflated. The vascularity of the lower limb was assessed immediately and after 30 minutes. The assessment was conducted through clinical palpation of pulses, pin-prick, and SpO_2_ measurement at the great toe. The vascularity was satisfactory, with no signs of ischemia. Flap harvest proceeded as planned.

**Figure 4:**
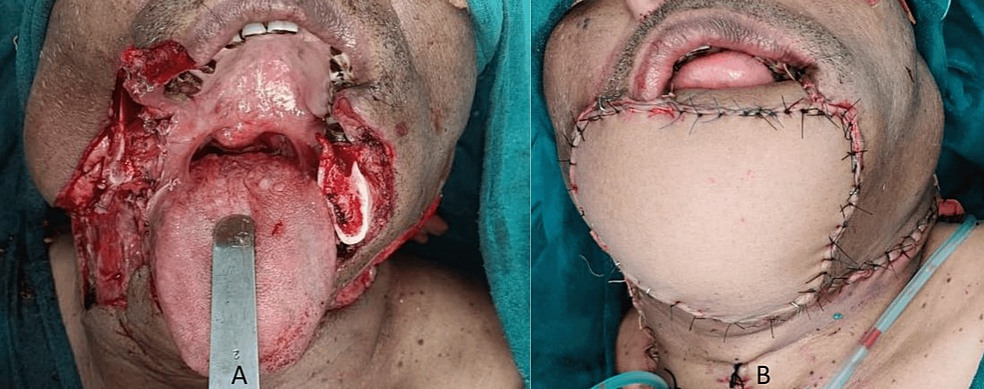
Intraoperative images A: Surgical defect after the tumor removal. B: Final reconstruction

The patient underwent composite resection of the lesion with mid-segmental mandibulectomy and bilateral MRND (Type III) (Figure 4A). The harvested fibula bone was osteotomized to provide a 3 cm segment for the right body, 2.5 cm for the central segment, and 7 cm for the left body of the mandible. Anastomosis to neck vessels followed by a free ALT flap for skin defect reconstruction was performed (Figure 4B).

**Figure 5:**
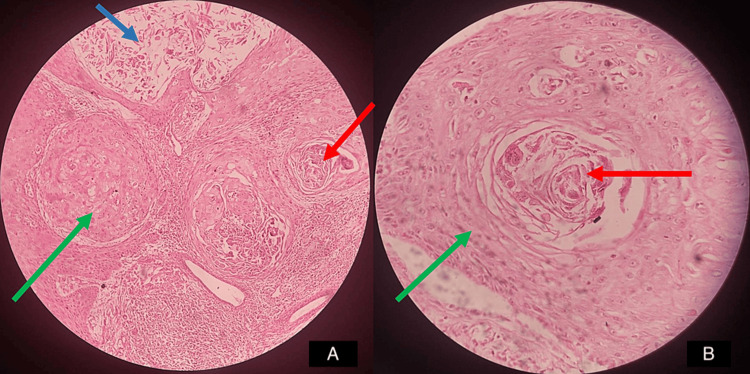
Microscopic image of the resected specimen (tumor) A: Low-power view. Hematoxylin and eosin (H&E)-stained section depicting stratified squamous epithelium and connective tissue. The section shows epithelial islands within a dense connective tissue stroma. The epithelium displays keratin pearls, and the underlying connective tissue exhibits blood vessels, fibroblasts, and collagen fibers. B: High-power view, showing the epithelium and keratin pearl in detail

The histopathological examination (HPE) revealed a moderately differentiated squamous cell carcinoma with negative margins (Figure 5A, 5B).

**Figure 6:**
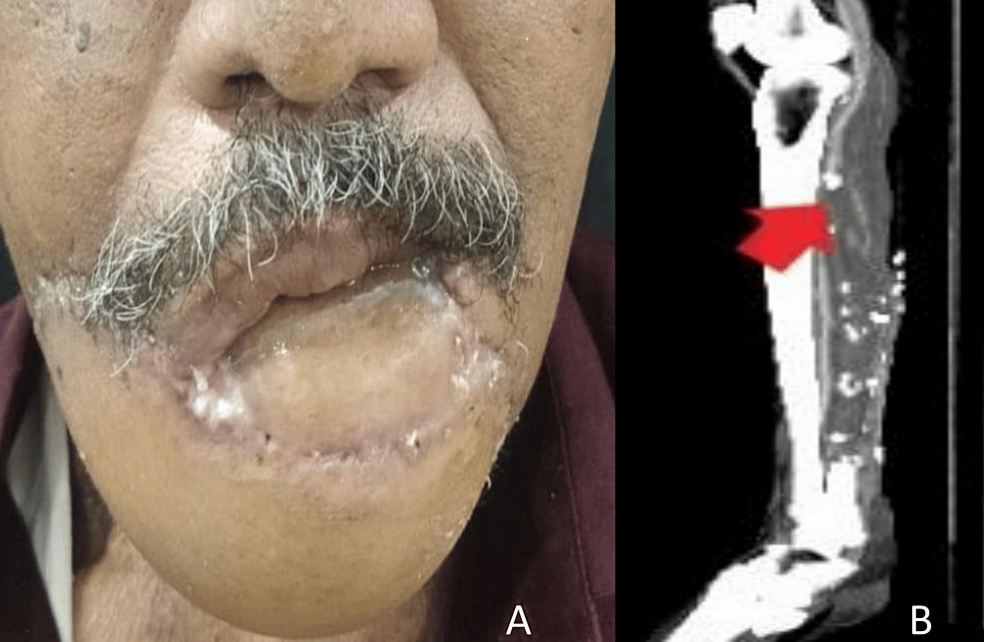
Postoperative images A: Follow-up image at six months postoperatively. B: CT angiogram of the left lower limb shows the sagittal section showing hypoplastic PTA PTA: posterior tibial artery

Subsequently, the patient was advised adjuvant chemoradiotherapy. Three months postoperatively, there were no reported ischemic events or claudication in the left leg. The flap maintained viability without any recurrence of the tumor (Figure 6A). A postoperative CT angiogram was conducted in which the sagittal section revealed hypoplastic PTA (Figure 6B). There were no short-term or long-term donor site complications.

## Discussion

The FFF is unique in its ability to reconstruct large bony defects while providing flexibility for multiple osteotomies. Blood supply to the lower limb primarily comes from the anterior tibial artery (ATA), PTA, and PA. Furthermore, the main popliteal artery and its branches near the knee, including the lateral and medial inferior geniculate arteries, anterior recurrent tibial artery, and medial and lateral sural arteries, also play a role in supplying blood to the leg [4]. The FFF relies on the PA for its vascular supply, but the infra-popliteal arterial vasculature can be subject to variations. Such variations are classified by Kim-Lippert's classification [5]. The Type III variations describe hypoplasia or aplasia of one or more main vessels of the leg. In Type IIIA in arterial Kim-Lippert's classification of infrapopliteal branching, the PTA may be hypoplastic or aplastic. This variation is relatively rare, with a prevalence of approximately 3.3%. The other two types are Type IIIB (1.5%), where the ATA may be hypoplastic or aplastic, and Type IIIC (0.4%), also called peronea arteria magna, where both the PTA and ATA may be hypoplastic or aplastic. There have been relatively few reported cases where an incidental intraoperative finding of the Type IIIA variation led to a successful FFF outcome.

The use of preoperative vascular mapping before FFF transfer remains a topic of debate. Proponents of preoperative vascular mapping argue that it allows for a thorough understanding of the vascular anatomy, including any variations that may impact the success of FFF transfer. This knowledge can help surgeons anticipate and mitigate potential complications during the procedure, improving patient outcomes. On the other hand, critics of routine preoperative vascular mapping point to the additional time and resources required for this step, which may not always be feasible in resource-constrained and emergent cases. The argument is that sufficient clinical and intraoperative adaptation to anatomical variations may reduce the need for preoperative mapping.

Routinely, we visually locate the ATA and PTA before the division of the distal PA. In cases where the PTA is hypoplastic or aplastic, the vascularity of the limb can be ascertained conclusively by placing clamps over the proximal and distal PAs. The fibula can be safely harvested only after clinically confirming the vascularity of the leg with clamps in place. Similar findings were echoed by Kumar et al. [6], Oxford and Ducic [7], Golas et al. [8], and Lutz et al. [9] in their respective studies. Taylor and Pan [10] noted that most leg muscles are supplied by two or three blood vessels, with extensive connections among all leg vessels. These connections can compensate for reduced blood supply if one or two major arteries become blocked, ensuring perfusion to the remainder of the leg. This mechanism explains the well-preserved perfusion of the leg in cases of Type IIIA anomalies following fibula harvest.

## Conclusions

We conclude that the FFF harvest can be successfully conducted in cases of Type IIIA infra-popliteal vascular variation by confirming the lower limb vascularity after clamping the proximal and distal PAs. Additionally, in patients with unremarkable clinical examinations of distal pedal pulses, routine preoperative imaging to assess the vascularity of the donor limb for the FFF is unnecessary.

## References

[1] Taylor GI, Miller GD, Ham FJ (1975). The free vascularized bone graft. A clinical extension of microvascular techniques. Plast Reconstr Surg.

[2] Cordeiro PG, Disa JJ, Hidalgo DA, Hu QY (1999). Reconstruction of the mandible with osseous free flaps: a 10-year experience with 150 consecutive patients. Plast Reconstr Surg.

[3] Kim D, Orron DE, Skillman JJ (1989). Surgical significance of popliteal arterial variants. A unified angiographic classification. Ann Surg.

[4] Bowers Z, Nassereddin A, Sinkler MA, Bordoni B (2023). Anatomy, bony pelvis and lower limb: popliteal artery. StatPearls [Internet].

[5] Abou-Foul AK, Borumandi F (2016). Anatomical variants of lower limb vasculature and implications for free fibula flap: systematic review and critical analysis. Microsurgery.

[6] Kumar V, Gupta PK, Bindu A, Mantri M, Mathews S, Jaiswal D, Kant Shankhdhar V (2023). Safety of free fibula flap harvest in IIIA and IIIB tibio-peroneal trunk variations. J Plast Reconstr Aesthet Surg.

[7] Oxford L, Ducic Y (2005). Use of fibula-free tissue transfer with preoperative 2-vessel runoff to the lower extremity. Arch Facial Plast Surg.

[8] Golas AR, Levine JP, Ream J, Rodriguez ED (2016). Aberrant lower extremity arterial anatomy in microvascular free fibula flap candidates: management algorithm and case presentations. J Craniofac Surg.

[9] Lutz BS, Wei FC, Ng SH, Chen IH, Chen SH (1999). Routine donor leg angiography before vascularized free fibula transplantation is not necessary: a prospective study in 120 clinical cases. Plast Reconstr Surg.

[10] Taylor IG, Pan WR (1998). Angiosomes of the leg: anatomic study and clinical implications. Plast Reconstr Surg.

